# Improving standards for reporting studies involving humans and experimental animals in the *British Journal of Nutrition* and in the *Journal of Nutritional Science*

**DOI:** 10.1017/jns.2014.50

**Published:** 2014-09-15

**Authors:** Graham C. Burdge

**Affiliations:** Editor-in-Chief of the *British Journal of Nutrition* and of the *Journal of Nutritional Science*, Academic Unit of Human Development and Health Faculty of Medicine, University of Southampton, Southampton, UK email g.burdge@nutritionsociety.org

Accurate reporting of research methods is critical for the quality, reliability and integrity of scientific research. Inadequate reporting of experimental methods may lead to publications that are of limited value as a source of scientific evidence on which to base future research or to inform policy or health practice^(^^1^^)^. As a consequence, such articles represent considerable waste in research funding and endeavour^(^^2^^)^. To improve the quality of reporting of research methods in the *British Journal of Nutrition* (*Br J Nutr*) and in the *Journal of Nutritional Science* (*J Nutr Sci*), two amendments to the requirements for publication in these journals will be implemented during 2014. These will address specifically studies involving experimental animals and the reporting of randomised controlled trials (RCT) in human subjects.

A survey that was commissioned by the National Centre for the Replacement, Refinement and Reduction of Animals in Research (NC3Rs) that assessed 271 articles reporting studies involving experimental animals that were carried out in the UK and in the USA identified serious under-reporting of research methods^(^^1^^)^. These included a lack of a stated hypothesis (41%), no apparent randomisation between treatments (87%), researchers were not blinded to the allocation of animals to different experimental groups (86%), and no description of statistical methods or no reporting of between-subject variation (30%). To improve the reporting of experimental methods in articles that describe studies involving animals, NC3Rs have developed the Animal Research: Reporting *In Vivo* Experiments (ARRIVE) guidelines that are captured in a twenty-item checklist describing the minimum standards for reporting such experiments^(^^1^^,^^3^^–^^6^^)^. These guidelines are detailed on the NC3Rs website (www.nc3rs.org.uk/ARRIVE) where the checklist is available freely in English, Chinese, Italian and Portuguese. As stated in the current Instructions for Contributors, these guidelines have been adopted by the *Br J Nutr* and by the *J Nutr Sci*. It is now a requirement that all manuscripts submitted to these journals that describe studies involving experimental animals meet these guidelines as a minimum standard of reporting. Manuscripts that do not meet these standards will not be considered for publication.

Incomplete or inaccurate reporting of experimental methods has also been reported in a number of reviews of RCT involving human subjects^(^^7^^–^^12^^)^. Such inadequate reporting may obscure deficiencies in experimental design that could, for example, bias the study outcome or exaggerated treatment effects^(^^13^^)^, which, in turn, could mislead health policy decisions as well as undermine the quality of the scientific evidence base. Although these reviews were based upon surveys of clinical trials involving medicines, the same principles apply to RCT in human nutrition, particularly in the context of associations between diet and health outcomes. Guidelines for best practice in the design and conduct of dietary intervention trials in human subjects have been proposed by two recent articles^(^^14^^,^^15^^)^. These articles emphasise the importance of accurate reporting of the study design and recommend adherence to the Consolidated Standards of Reporting Trials (CONSORT) guidelines^(^^13^^)^ (www.consort-statement.org). To promote best practice, manuscripts reporting RCT involving human subjects will be required to demonstrate compliance with the CONSORT guidelines. This requirement includes the registration of the trial with the appropriate authority and will apply to dietary intervention or lifestyle studies and to studies that involve acute dietary change, for example, modification of a meal as a part of a postprandial metabolism study. The date on which compliance with the CONSORT guidelines will be implemented will be announced in advance on the respective manuscript submission webpages (http://mc.manuscriptcentral.com/bjn; http://mc.manuscriptcentral.com/jns). After this date, manuscripts that report RCT which do not comply with this requirement will not be considered by the *Br J Nutr* or by the *J Nutr Sci*.

I hope that these changes to the requirements for publication in the *Br J Nutr* and in the *J Nutr Sci* will not be considered onerous by authors, particularly as many of the principles of the ARRIVE and CONSORT guidelines are captured in the current Instructions for Contributors. Adoption by the *Br J Nutr* and in the *J Nutr Sci* of these widely recognised standards for reporting studies will align these journals with or exceed those of similar journals in the field, thus maintaining their reputation for publishing high quality research in nutritional science.
